# Validation of a visual analogue scale for the evaluation of the postoperative anxiety: A prospective observational study

**DOI:** 10.1002/nop2.330

**Published:** 2019-07-11

**Authors:** François Labaste, Fabrice Ferré, Hélène Combelles, Valentin Rey, Jean‐Christophe Foissac, Anne Senechal, Jean‐Marie Conil, Vincent Minville

**Affiliations:** ^1^ Department of Anesthesiology and Intensive Care University Hospital of Toulouse Toulouse France; ^2^ Institut des Maladies Métaboliques et Cardiovasculaires, INSERM U1048 Université de Toulouse, UPS Toulouse France

**Keywords:** anxiety, nurse evaluation, pain, Spielberger, STAI, visual analogue scale

## Abstract

**Aim:**

Anxiety affects the perception of pain during the postoperative period. A simple evaluation scale could improve the management of this component. The objective of this study was to evaluate the reproducibility and the consistency of a visual analogue scale for anxiety compared with the reference method, the State‐Trait Anxiety Inventory (STAI).

**Design:**

Observational, prospective, monocentric study of 500 patients in the post‐anaesthetist care unit. Anxiety was evaluated using both the visual analogue scale for anxiety and the STAI in perioperative patients. Consistency between the visual analogue scale for anxiety and the STAI, detection thresholds and factors predicting anxiety were researched.

**Results:**

A correlation was found between the visual analogue scale for anxiety and the STAI. There was also a correlation between pain and anxiety. Analysis of receiver operating characteristic (ROC) curves showed a visual analogue scale for anxiety threshold of 34/100 allowing the identification of patients with or without anxiety. Predictive factors for anxiety are female gender, use of benzodiazepine in premedication, emergency surgery and significant pain in the post‐anaesthetist care unit. In summary, visual analogue scale for anxiety is a useful tool for detecting the anxiety component of postoperative pain. It could be used in association with covariates of interest to improve anxiety management during the postoperative period.

## 1 BACKGROUND

The evaluation and management of pain is a necessary requirement in evolved health systems and constitutes a real challenge in public health. The law in France relating to the rights of the sick and the quality of the health system recognizes the relief of pain as a fundamental right. Pain is defined as “an unpleasant sensory and emotional experience associated with actual or potential tissue damage or described in terms of such damage” (IASP). This definition thus recognizes a psychopathological aspect to pain of which the most prevalent trait is anxiety. Pain and anxiety are closely interlinked; the latter modifies patients’ perceptions, accentuates postoperative pain and affects their quality of life and long‐term outcome (Cornwall & Donderi, 1988; Ploghaus et al., 2001). Pain and anxiety involve a complex combination of fear, apprehension, agitation and feeling of malaise associated with physical manifestations (Dunn et al., 2018). Pain management would therefore benefit from a multidimensional evaluation procedure. Patients are instrumental in this management, and their participation is therefore essential in the evaluation of the intensity of their symptoms. The VAS self‐evaluation scale, first described by Aitken (1969), is the scale currently most used to analyse the pain aspect. It is simple, rapid and does not require a high level of skill. The “anxiety” aspect is usually assessed using a standard scale, the STAI described by Spielberger et al. in Consulting Psychologists (1970) and Lemche, Chaban, and Lemche (2016). The STAI is composed of forty questions, but is time‐consuming, unwieldy, difficult to implement and not adapted to everyday clinical practice (Lemche et al., 2016). The concept of a VAS adapted to measure anxiety (the VAS‐A) was proposed in 1976 and used for the first time in 1988 for a small series of patients undergoing dental treatment (Luyk, Beck, & Weaver, 1988). This scale has since been validated in several studies of weak methodological quality (Berghmans et al., 2017; Facco et al., 2013). Up to this point, no study has been concerned with the postoperative care of adult patients.

The principal objective of this study was to assess the validity and reliability of the VAS‐A in comparison with the STAI in the evaluation of anxiety of patients during the immediate postoperative period. The secondary objective was to determine, during the course of the same period, the factors associated with postoperative anxiety, in particular the relationship between anxiety and pain.

## 2 MATERIALS AND METHODS

### 2.1 Plan of study

We conducted a prospective observational study in a single centre. The study was approved by the Research Ethics Committee of Toulouse University Hospitals (no. 60‐0714). As the study was non‐interventional, written patient consent was not collected. Nonetheless, it was anticipated that data derived from routine patient care would not be collected in the case of patients or their representatives expressly refusing inclusion in the study. Data collected were strictly anonymous, and in no case were the data transmitted to any individual other than the principal investigator.

### 2.2 Study population

A total of 500 patients were included postoperatively after emergency and elective surgery (urological, visceral, plastic, gynaecological, vascular and cancer). Patients had to be 18 years or more old and to present cognitive capacities and language competence sufficient to respond to the questionnaire and must not have opposed their participation in the study. Patients presenting difficulties in judgement or comprehension, language problems, visual impairment or incapacity to move their upper limbs were excluded from the study.

### 2.3 Data collection

Data were collected prospectively for each patient, in a standardized manner using a data collection form by an examiner not involved in patient care, trained and experienced in using different scales (data collected by HC, VR, JCF and AS). Anxiety levels were assessed using both the VAS‐A and the STAI fifteen minutes after admission to the post‐anaesthetist care unit (PACU), then a second time after authorization for discharge from the PACU. The VAS‐A scale is comprised of a horizontal line 100mm long with the indication “no anxiety” to the left and “worst possible anxiety” to the right (see appendix, no copyright). A verified French translation of the A‐State STAI Form Y was used. The A‐State scale relates uniquely to what the subject feels at the time of taking the inventory, as opposed to the A‐Trait scale, which is designed to assess what the subject feels generally. Form was designed to remove inventory items linked more to depression. The examiner who put the list of questions to the patients obtained the STAI score. Each patient was required to respond to the 20 questions using responses, which were rated from 1–4 (Likert‐type scale). We considered a STAI score > 40 as evidence of a state of anxiety. Possible scores varied between 20–80. The scales were used in the same order for each patient. A double evaluation of pain was also undertaken using both the VAS and the NRS at PACU admission and discharge. Information concerning patient characteristics, surgical intervention, type of anaesthetic, premedication and treatments was obtained through oral questioning and the anaesthetic record. This evaluation did not interfere with normal patient care and did not dictate anxiolytic treatment.

### 2.4 Statistical analysis

Demographic data and pain and anxiety scores were abstracted and described through descriptive statistical analysis. A study of the distribution of the values was carried out using a Kolmogorov–Smirnov test, with, in parallel, analyses of the coefficient of kurtosis and the coefficient of skewness. Results were expressed as the median and confidence interval CI 95% [ ] for quantitative variables and in numbers and percentages ( ) for qualitative variables.

The study population was separated into two groups: non‐anxious (STAI score < 40) and anxious (STAI score > 40) in respect of each of two time points: admission to and discharge from the PACU. Patient characteristics for the two groups were compared using:

- Non‐parametric tests (Mann–Whitney *U* test) for continuous variables, because of the non‐homogeneity of the groups and the non‐Gaussian distribution of most variables; and
- Fisher's exact test for qualitative variables.

The discrimination thresholds of the VAS‐A and pain evaluation scales at PACU admission and discharge for anxious or non‐anxious character were determined by reference to ROC curves and their related AUC. The choice of the optimal discrimination thresholds was made according to the best Youden's index. In parallel PCP, PCN, sensitivity and specificity were calculated according to these thresholds. Cannesson's two‐step procedure was used to determine a grey zone (or zone of uncertainty) for each threshold. The ROC curves were then compared so that the covariates with the least discriminatory power could be deleted. Related variables were identified with the help of a correlation table and a correlogram, using Spearman's rank correlation coefficient (Spearman's rho). Calculation of the correlation between the anxiety and pain scales was completed by a consistency study by analysing the intraclass correlation coefficient (ICC). In a final stage, the relationship between the different covariates and the explained variable (anxiety) was evaluated by multivariate analysis (logistic regression) using an *odds ratio* measure. We used a backward elimination procedure, which involved starting with all the variables with *p* < 0.2, then progressively deleting those that were not statistically significant. The Hosmer–Lemeshow adequacy test for *chi‐square goodness of fit* was used to choose the model for which the data were best adjusted. The study was carried out using MedCalc® statistical software version 15 (Mariakerke, Belgique). A *p* < 0.05 was considered statistically significant.

## 3 RESULTS

**Figure 1:**
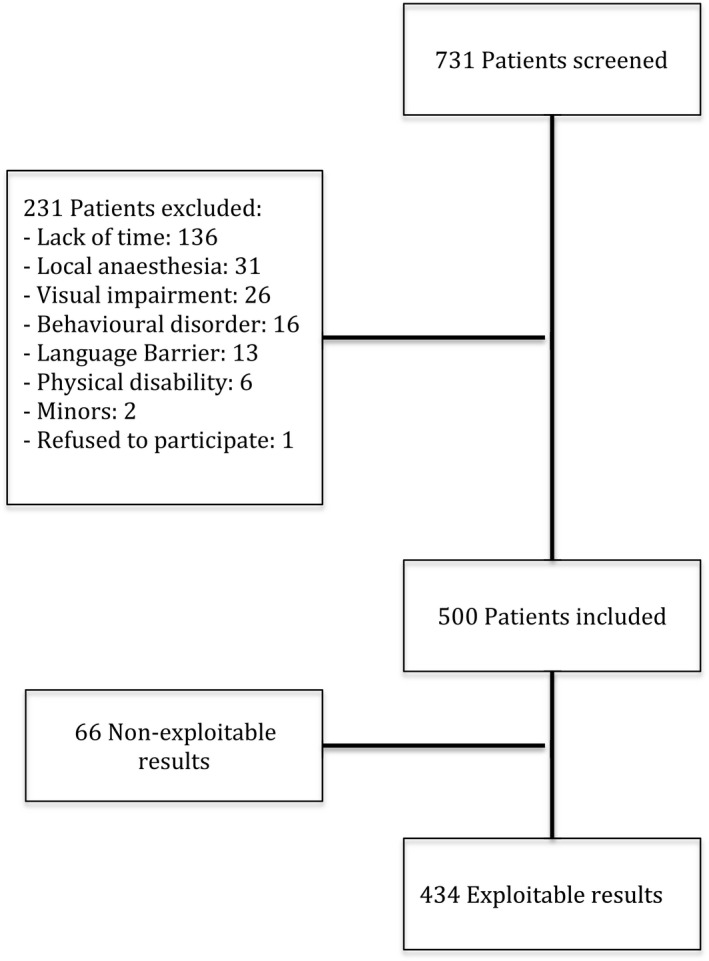
Flow chart

A total of 731 patients were admitted to the PACU during the period of the study. Among the 500 patients included, 66 were unable to be included because of too great pain or asthenia or failure to understand one or more evaluation tools (Figure 1). The demographic data for the whole population, the anxious group and non‐anxious group at PACU admission and discharge are set out in Table 1. Nine‐point two per cent of patients were anxious on admission to the PACU and 5.8% at discharge, according to the STAI. At admission to the PACU, there were more women (70% vs. 47% *p* = 0.07) and more analgesic treatments administered (80% vs. 55% *p* = 0.002) in the anxious group. At discharge from the PACU, there were more patients who had had benzodiazepines in PM (61% vs. 39% *p* = 0.029), more patients who had undergone emergency surgery (18% vs. 6% *p* = 0.038) and more analgesic treatments administered (89% vs. 58% *p* = 0.001) in the anxious group. The VAS scores were significantly higher at admission (51[40; 66] vs. 24[20; 29.5]) and discharge (41[34.1; 50] vs. 23[20; 26]) in the anxious group. This was also the case with regard to NRS scores.

**Table 1:** Patient characteristics

Items Median [95% CI]	Total	Non‐anxious Admission PACU	Anxious Admission PACU	*p*	Non‐anxious Discharge PACU	Anxious Discharge PACU	*p*
Age (years)	54 [53–57]	54 [52–57]	47 [40.3–58.3]	0.0617	54 [52–57]	52 [40.7–63.6]	0.3175
BMI (kg/m^2^)	24.75 [24.2–25.2]	24,8 [24.4–25.3]	24.1 [22.3–26.8]	0.629	25 [24.4–25.5]	23.5 [21.4–25.2]	0.073
ASA	2 [2–2]	2 [2–2]	2 [2–2]	0.6518	2 [2–2]	2 [2–2]	0.4424
VAS admission PACU	15 [11–20]	24 [20–29.5]	51 [40–66]	**<0.0001**			
VAS discharge PACU	10 [5–12]				23 [20–26]	41 [34.1–50]	**<0.0001**
NRS admission PACU	4 [4–4]	3 [3–4]	6.5 [5–7]	**<0.0001**			
NRS discharge PACU	3 [3–3]				3 [3–3]	5 [4–6]	**<0.0001**
Sex							
Female/male	49/51	47/53	70/30	**0.007**	48/52	54/46	0.567
Smoker							
Yes/no	22/78	22/78	22/78	1.000	22/78	28/72	0.490
First time surgery							
Yes/no	5/95	6/94	0/100	0.253	6/94	0/100	0.387
PM							
Yes/no	76/24	76/24	75/25	0.847	76/24	79/21	0.999
Benzodiazepines PM							
Yes/no	40/60	39/61	45/55	0,501	39/61	61/39	**0.029**
Antihyperalgesic							
	28/72	28/72	25/75	0.715	29/71	43/57	0.138
Type of anaesthesia							
LRA/GA	7/93	7/93	0/100	0.095	6/94	0/100	0.392
Type of anaesthetic							
Halogen/TIVA	89/11	89/11	85/15	0.414	87/13	78/21	0.249
Emergency surgery							
Yes/no	7/93	7/93	10/90	0.520	6/94	18/82	**0.038**
Cancer surgery							
Yes/no	11/89	11/89	15/85	0.438	12/88	25/75	0.075
Analgesics in PACU							
Yes/no	57/43	55/45	80/20	**0.002**	58/42	89/11	**0.001**

Bold values signifies P < 0.05.

**Figure 2:**
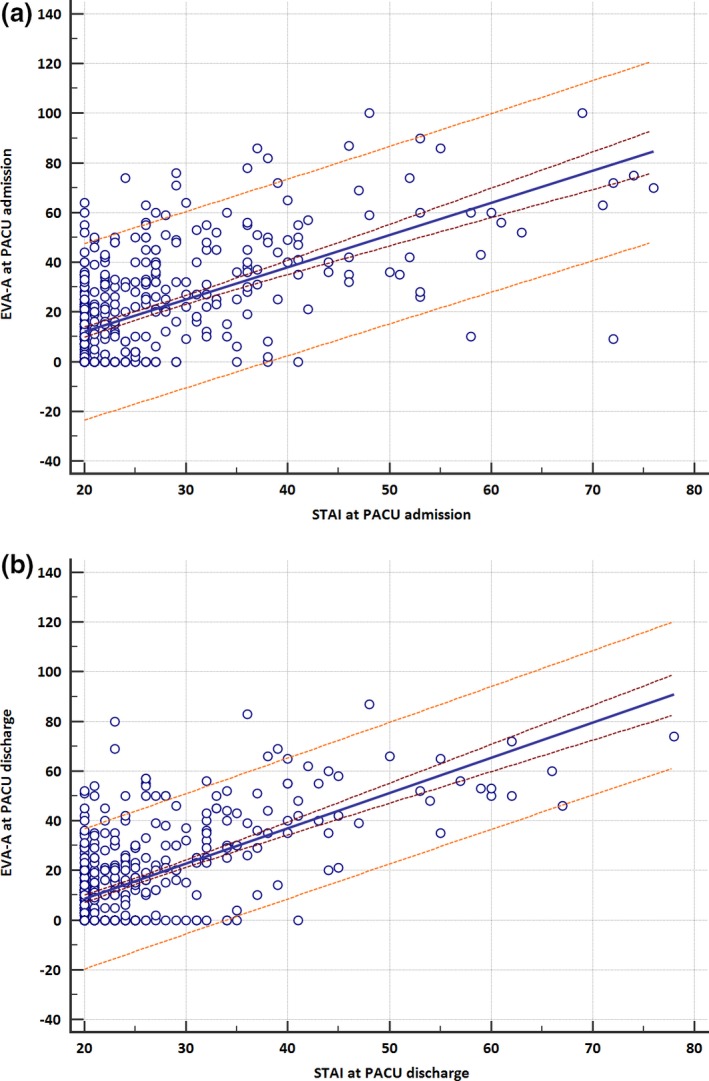
Correlation of evaluation scale scores VAS‐A and STAI (a: PACU admission, b: PACU discharge)

**Figure 3:**
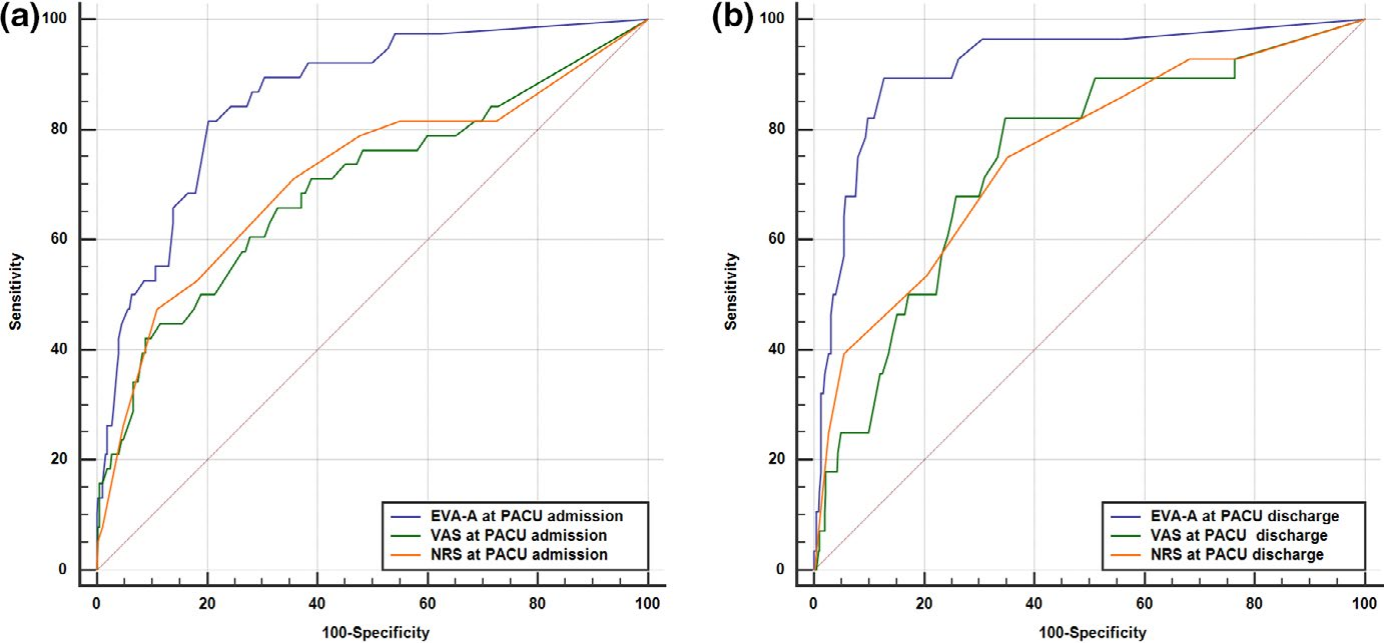
Comparison of ROC curves for VAS‐A and pain scales in anxious groups (STAI > 40) at PACU admission (a) and discharge (b)

Anxiety was significantly higher at admission to PACU than at discharge with VAS‐A score (15[11; 20] vs. 10[5; 12]) and STAI score (22[22; 23] vs. 21[20; 21]) (Table 2). Gender was a determining factor in anxiety and pain scores. Scores for women were significantly higher with a VAS‐A on admission to the PACU of (22[18; 6] vs. 8[3; 12] *p* = 0.0002), a VAS on admission of (39[32; 42] vs. 20[12; 24 0.9] *p* < 0.0001) and a VAS score on discharge from the PACU of (27.5[2; 30] vs. 21[18; 23.9] *p* = 0.023). The VAS‐A presented a statistically significant correlation with the STAI on admission to the PACU (*r* = 0.555) and on discharge from the PACU (*r* = 0.593) (Figure 2a,b). A correlation also existed between the NRS and VAS scores for evaluation of pain at admission to the PACU (*r* = 0.866) and at discharge (*r* = 0.873). The analysis using ROC curves of the discrimination thresholds of the VAS‐A and the pain evaluation scales according to anxious character is set out in Table 3. Comparison of the ROC curves (Figure 3a,b) demonstrated the superiority of the VAS‐A in detecting anxiety both on admission to and discharge from the PACU with a threshold of 34 (identical for admission and discharge). Only the VAS‐A (AUC > 0.85) has demonstrated a strong discriminatory power with acceptable grey zones and adequate sensitivity–specificity. The PCP of the threshold, however, remained <50%. The pain evaluation scales had insufficient discriminatory power, statistically much lower than that of the VAS‐A, as shown in Figure 3a,b, with an AUC greater at admission (*p* = 0.0023 for AUC VAS‐A vs. VAS and *p* = 0.011 for AUC VAS‐A vs. NRS) than at discharge from the PACU (*p* = 0.0001 for AUC VAS‐A vs. VAS et *p* = 0.0004 for AUC VAS‐A vs. NRS). The examination of the ICC is described in Table 4. This consistency test showed a moderate to good reproducibility of the VAS‐A in comparison with the STAI. (Reproducibility was considered excellent where ICC > 0.75 and moderate to good where ICC was between 0.4–0.75.). The analysis of the relationship between the different covariates and the explained variable (anxiety) using multivariate analysis (logistic regression) is set out in Table 5. The covariates relating to anxiety at admission to the PACU were centred by a VAS at admission of >62 and gender; men appear to be more protected from anxiety than women. On application of the adjustment test to this model, the critical value for *p* was only 0.6, but none of the other models tested were as well adjusted. Furthermore, the percentage of cases correctly classified by this model was 90.59% and the AUC value was 0.784 [CI 95% = 0.74–0.82]. The covariates relating to anxiety at discharge from the PACU were found to include a VAS cut‐off value >30 (the cut‐off value for pain at discharge calculated by the ROC curve), but also the fact that the surgery performed was emergency surgery, as well as the use of benzodiazepines in PM. The Hosmer–Lemeshow adequacy test showed an excellent adjustment of 0.82 as well as a percentage of cases correctly classified of 94%. The model was sufficiently discriminatory with an AUC of 0.83 [CI 95% = 0.79–0.86].

**Table 2:** Anxiety evaluation scale scores for entire study population

Scale	Admission PACU Median [95% CI]	Discharge PACU Median [95% CI]	*p*
STAI	22 [22–23]	21 [20–21]	<0.0001
VAS‐A	15 [11–20]	10 [5–12]	<0.0001

**Table 3:** Scale characteristics

	AUC	cut‐off	Grey Zone	Se. %	Sp. %	PCP %	PCN %
VAS‐A							
VAS‐A admission	0.8618	>34	21–47	81.58	79.95	29.2	97.7
VAS‐A discharge	0.9152	>34	21–37	89.29	87.33	30.5	99.2
VAS							
VAS admission	0.698	> 62	0–69	42,11	91,22	32,7	94,0
VAS discharge	0.75	>30	1–50	82	65	13	98
NRS							
NRS admission	0.724	>6	0–6.2	50	89	32	95
NRS discharge	0.758	>3	1.4–4.7	75	65	12	98

**Table 4:** Intracoefficient class between VAS‐A and STAI

	ICC	95% confidence interval
VAS‐A and STAI admission PACU		
Single measures	0.4251	0.3186–0.5172
Average measures	0.5966	0.4833–0.6818
VAS‐A and STAI discharge PACU		
Single measures	0.4018	0.1613–0.5691
Average measures	0.5733	0.2778–0.7253

**Table 5:** Multivariate analysis of predictive factors for anxiety

	*p*	OR	[CI 95%]
Anxiety at PACU admission			
VAS admission > 62	<0.0001	5.647	2.66–12
Gender; M = 1 ‐ F = 0	0.0276	0.416	0.19–0.91
Anxiety at PACU discharge			
VAS discharge PACU > 30	<0.0001	11.55	4.05–32.97
Emergency surgery	0.0003	11.95	3.16–45.2
Benzodiazepines in PM	0.0045	3.763	1.51–9.39

## 4 DISCUSSION

The objective of this study was to assess the validity and reliability of the VAS‐A in comparison with the STAI in the assessment of postoperative anxiety in adults. Among the 500 participants in the study, only a small number (9.5%) presented an anxious state on admission to the PACU using the reference method, that is the STAI. The cut‐off chosen to separate anxious from non‐anxious patients using the STAI was 40. This threshold remains debatable. The average scores obtained in the general French population are 37 for men and 42 for women (depending on age). While in some studies a STAI threshold > 45 was used (Kindler, Harms, Amsler, Ihde‐Scholl, & Scheidegger, 2000; Millar, Jelicic, Bonke, & Asbury, 1995) , in most studies a pooled score of 40 was chosen (Facco et al., 2013).

In relation to the study population, a good to moderate statistical correlation between VAS‐A and STAI, (*r* = 0.555 at admission to and 0.593 at discharge from the PACU) and a moderate predictability were found. These values are close to those found in most works where the correlation coefficient r observed varied between 0.50–0.65. (Bringuier et al., 2009; Chlan, Savik, & Weinert, 2003; Kindler et al., 2000; Millar et al., 1995) These concerned either study with a small number of patients (<100) including solely women or children or studies, which did not take preoperative anxiety into account. A correlation coefficient greatly superior to the others was found in one single study in outpatient surgery patients with *r* = 0.82 (Vogelsang, 1988).

For a VAS‐A threshold of 34 in the population of this study, sensitivity (82%), specificity (80%) and PCV (98%) were good, but the PCP was low (29%). These values are close to those found in other writings (46% Facco; 30% Capdevilla; 30% Kindler; 34% Millar; 20% Kindler) (Bringuier et al., 2009; Facco et al., 2013; Kindler et al., 2000; Millar et al., 1995). The VAS‐A is more sensitive and discriminating than either the NRS or the VAS in detecting anxious patients. The measures were taken at two points during care (test–retest method). There is therefore as regards the VAS‐A both a good reproducibility and a good ability to detect rapid changes in anxiety levels.

The univariate analysis demonstrated several factors relating to anxiety, firstly, the female gender, the most statistically significant factor and confirmed in numerous studies (Badner, Nielson, Munk, Kwiatkowska, & Gelb, 1990; Domar, Everett, & Keller, 1989; Graham & Conley, 1971; Kindler et al., 2000; Luyk et al., 1988; van Wijk & Smalhout, 1990). The use of benzodiazepines in PM is also a predictive factor for anxiety. A study found a statistically significant correlation between the consumption of benzodiazepines and anxiety (Kindler et al., 2000). This can be explained by a paradoxical effect of benzodiazepines which however is very uncommon (<1%) (Mancuso, Tanzi, & Gabay, 2004), namely their effect on the memory (anterograde amnesia) which causes delirium in the PACU (Lepousé, Lautner, Liu, Gomis, & Leon, 2006). Several recent studies have moreover demonstrated that benzodiazepines have no advantage over placebos in PM (Abdul‐Latif, Putland, McCluskey, Meadows, & Remington, 2001; Beydon et al., 2015; Maurice‐Szamburski et al., 2015). The apparent correlation probably arises from a selection bias; patients considered to be anxious at the time of the preanaesthetic visit were given increased quantities of benzodiazepines in PM. Emergency surgery is associated with anxiety and even if that would appear logical, there is not to our knowledge any published report of an equivalent result. Analgesic treatment in the PACU is more statistically significant in the anxious group as found in published reports but also in the subgroup of women (Hsu et al., 2005; Johnson, Rice, Fuller, & Endress, 1978; Martinez, Fletcher, Bouhassira, Sessler, & Chauvin, 2007; Nielsen, Nørgaard, Rasmussen, & Kehlet, 2007; Pan et al., 2006; Strulov et al., 2007; Taenzer, Melzack, & Jeans, 1986).

Finally, in multivariate analysis, the factors, which condition postoperative anxiety on admission to the PACU, were the female gender and a VAS > 62. The factors relevant to anxiety on discharge from the PACU are the use of benzodiazepines in PM, emergency surgery and a VAS > 30. Taenzer also found a statistically significant correlation between the VAS score and the level of anxiety without however having determined a precise threshold (Bisgaard, Klarskov, Rosenberg, & Kehlet, 2001; Taenzer et al., 1986; Werner, Mjöbo, Nielsen, & Rudin, 2010).

Points of methodology are open to argument notably that the order of use of the two scales could influence results. The use of STAI first could affect the mood of the patient and therefore influence the patient's response to the VAS‐A, thus making it potentially desirable to randomize the order in which the scales are used. The bias was diminished because a single examiner trained and experienced in the use of the two scales made the readings. The items on the STAI were read out and filled in by the examiner, even though the scale is valid as self‐evaluation. However, in a postoperative context, most patients were unable to complete the questionnaire without help.

The VAS‐A is a means of measurement less precise because it contains fewer questions in comparison with the STAI (Likert‐type scale of 40 questions) but it proves to be above all more rapid and simple (Lemche et al., 2016). It is thus a more global and multidimensional means for evaluating anxiety. The VAS‐A is probably a less familiar technique as it is non‐verbal and without quantifiable values. When using a VAS, patients avoided the extreme values and had a tendency to give responses towards the centre of the scale (Poulton, 1979,1982). On the other hand, the distribution of values using the VAS‐A was non‐central. For the VAS‐A to be used as a tool to detect anxiety in the postoperative period, the other covariates relating to anxiety that were identified in this study should be integrated. This could be done by way of a score, on which our research group is currently working.

Our study has several limitations. First, phobic or anxious subjects by their very nature were not identified in our data collection. However, the aim of the study was to validate a scale applicable to all adult patients. Second, this is a single centre study. Thus, a validation of this result should be made in other centres. However, the sample size was large enough to be representative of the postoperative population.

In summary, the VAS‐A could be a useful tool for measuring postoperative anxiety. It allows detection of anxious patients on a score >34. Certain factors warn of a higher risk of anxiety: female gender, emergency surgery, use of benzodiazepines in PM and statistically significant pain in the PACU. We therefore advocate the use of this scale postoperatively in relation to adults to improve the management of anxious states in patients.

## CONFLICT OF INTEREST

This work should be attributed to the Department of Anesthesiology and Intensive Care, Toulouse University Hospital, Toulouse, France. Support was provided solely from institutional and department sources. Authors have not disclosed any potential conflicts of interest.
